# Phelan-McDermid syndrome-associated psychosis: a systematic review

**DOI:** 10.1017/neu.2024.46

**Published:** 2024-11-20

**Authors:** Mark A. Colijn

**Affiliations:** Department of Psychiatry, Hotchkiss Brain Institute, Mathison Centre for Mental Health Research and Education, University of Calgary, Calgary, AB, Canada

**Keywords:** Schizophrenia, psychotic disorders, genetics, neuropsychiatry, developmental disabilities

## Abstract

**Objective:**

Phelan-McDermid syndrome is a rare genetic disorder characterised by various neurodevelopmental, medical, and psychiatric issues. Although bipolar disorder-like presentations and catatonia are particularly common, psychosis has also been reported but is less well described. As such, this systematic review sought to characterise the phenomenology of psychosis in Phelan-McDermid syndrome, clarify the association of psychotic symptoms with other neuropsychiatric features of the disorder, and describe antipsychotic treatment response.

**Methods:**

A literature search was completed in July 2024 using PubMed and Scopus. Only English-language articles that reported the occurrence of psychotic symptoms in Phelan-McDermid syndrome were eligible for inclusion. 18 articles describing 35 individuals were included in the main analyses. Three additional articles of relevance are discussed separately, as they either provided limited clinical information or did not present data in a patient-specific manner.

**Results:**

The average age of psychosis onset was ∼17 years, and 65% of individuals developed symptoms at or before age 15. ∼69% of individuals also experienced catatonia, ∼81% experienced mood symptoms, and 50% experienced both. Visual hallucinations were the most commonly reported psychotic symptom. Where reported, ∼76% of individuals exhibited at least a partial and/or temporary response to antipsychotic therapy.

**Conclusion:**

Psychotic presentations in Phelan-McDermid syndrome may qualitatively differ from schizophrenia. Although numerous antipsychotics may be efficacious in the treatment of Phelan-McDermid syndrome-associated psychosis, this review most importantly highlights the paucity of available high-quality evidence to guide treatment decisions in this respect, and as such indicates the need for more reports to be published.

Summations

- Psychotic features that develop in the context of Phelan-McDermid syndrome commonly cooccur with catatonic and/or mood symptoms.
- The age of psychosis onset in Phelan-McDermid syndrome may be particularly early compared to general schizophrenia populations.
- Antipsychotic medications may be effective in treating psychosis in Phelan-McDermid syndrome.

Considerations

- Relatively few reports of psychosis in Phelan-McDermid syndrome have been published to date and the corresponding phenotypic information provided is often limited.
- In most reports of antipsychotic treatment response a mood stabilizer was concurrently used, complicating the interpretation of the therapeutic effects of each.
- The identification and characterisation of psychotic symptoms in Phelan-McDermid syndrome may be difficult given the common cooccurrence of intellectual disability and/or autism.

## Introduction

Phelan-McDermid syndrome is a rare genetic disorder caused by *SHANK3* haploinsufficiency, due to either a 22q13.3 deletion or a pathogenic *SHANK3* variant (Srivastava *et al*., 2023). Clinically, it is characterised by various developmental abnormalities (e.g., intellectual disability, autism, speech delays, and functional regression), congenital anomalies (e.g., cardiac and urogenital defects), and numerous other medical issues (e.g., epilepsy) (Srivastava *et al*., 2023). Psychiatric features are also common, including bipolar disorder-like presentations with rapid mood fluctuations, irritability/aggression, and significant sleep disruption, as well as catatonia (Srivastava *et al*., 2023). Although psychosis has additionally been reported, its association with Phelan-McDermid syndrome is comparatively less clear and prevalence estimates vary widely. For example, in their Phelan-McDermid syndrome cohort, Levy *et al*. (2022) found that approximately 4.5% of individuals had been diagnosed with schizophrenia or schizoaffective disorder, whereas psychotic symptoms were reported in 50% (19/38) of individuals in a study by Kohlenberg *et al*. (2020). Similarly, Dille *et al*. (2023) reported that five out of nine individuals in their study who went through the ‘neuropsychiatric decompensation’ stage of the disorder experienced hallucinations.

Only one systematic review in Phelan-McDermid syndrome to date specifically included an evaluation of psychotic symptoms (Kolevzon *et al*., 2019), and this paper, which was published approximately five years ago, identified only seven individuals who had experienced psychosis. Although numerous reports since have described the occurrence of psychotic symptoms in additional individuals with Phelan-McDermid syndrome, no up-to-date reviews on this topic exist. While expert consensus guidelines pertaining to the management of patients with Phelan-McDermid syndrome (Srivastava *et al*., 2023), including with respect to psychiatric issues in particular (van Balkom *et al*., 2023), have previously been published, these papers only briefly mention psychosis, without robustly reviewing the topic. As such, this systematic review sought to characterise the phenomenology of psychotic symptoms in Phelan-McDermid syndrome, their association with other neuropsychiatric features of the disorder, and their response to antipsychotic therapy.

## Methods

**Figure 1.**
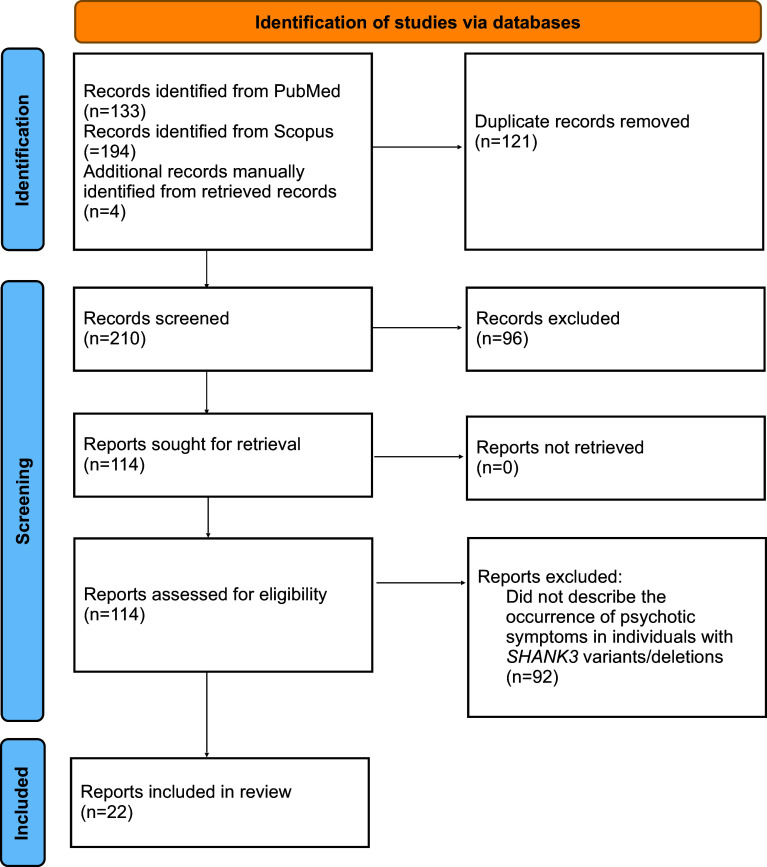
Literature search flow diagram for Phelan-McDermid syndrome-associated psychosis.

A literature search was completed in July 2024 using PubMed and Scopus. The terms ‘phelan mcdermid’, ‘phelan-mcdermid’, ‘PHMDS’, ‘SHANK3’, or ‘22q13’ were used in combination with ‘psychosis’, ‘psychotic’, ‘schizophrenia’, ‘schizoaffective’, ‘hallucination’, ‘delusion’, ‘paranoia’, or ‘paranoid’. No filters were used. The PubMed search yielded 133 results and the Scopus search yielded 194 results. After duplicates were removed 206 results remained. The search process is outlined in Fig. 1. Articles were manually screened by the author and only English-language articles that reported the occurrence of psychotic symptoms in humans harbouring (presumed) pathogenic variants associated with Phelan-McDermid syndrome (involving *SHANK3)* were eligible for inclusion. Studies of any methodology were eligible as long as the above inclusion criteria were met. Specific information extracted from each article (where available) included psychosis age of onset, history of catatonic symptoms, history of mood symptoms, the type of psychotic symptoms that occurred and/or any psychotic disorder diagnoses carried by the individuals, antipsychotic medications trialled (including daily dose, where available), antipsychotic treatment response, and any reported side effects. Reference lists of retrieved articles were manually reviewed by the author for any additional articles of relevance. OMIM was also reviewed. Four additional articles were identified as being eligible for inclusion as a result.

## Results

18 articles that described 35 individuals with Phelan-McDermid syndrome-associated genetic variants (affecting *SHANK3)* who experienced symptoms of psychosis were included in the main analyses (Denayer *et al*., 2012; Messias *et al*., 2013; Breckpot *et al*., 2016; Egger *et al*., 2016; Fokstuen *et al*., 2016, Li *et al*., 2016, Tabet *et al*., 2017; De Rubeis *et al*., 2018; Jungova *et al*., 2018; Accogli *et al*., 2019; Bey *et al*., 2020; Hu *et al*., 2020; Kohlenberg *et al*., 2020; Verhoeven *et al*., 2020; Galosi *et al*., 2021; Kankuri-Tammilehto *et al*., 2021; Rysstad *et al*., 2022; Boley *et al*., 2024). Relevant clinical information for these 35 individuals is summarised in Table 1. As noted in Table 1, while three individuals in the study by Verhoeven *et al*. (2020) had previously been described in the literature (Verhoeven *et al*., 2013; Egger *et al*., 2016), each is included only once in Table 1. While one additional article that described two siblings (one of whom had Phelan-McDermid syndrome) alluded to the occurrence of psychotic symptoms in both, in the actual case description of the sibling (sister) with Phelan-McDermid syndrome, there is no explicit mention of any frank psychotic symptoms (nor is there mention of a schizophrenia spectrum disorder diagnosis) (Huang *et al*., 2022). As such, this article is not included in Table 1, nor is it mentioned any further in this review.

**Table 1.** Patient-specific clinical information in Phelan-McDermid syndrome-associated psychosis

Study	Proband	Age of Psychosis Onset (years)	Catatonia	Comorbid Mood Symptoms	Psychotic Disorder Diagnosis and/or Psychotic Symptoms	Antipsychotic Therapy (total daily dose)^c^	Antipsychotic Treatment Response	Side Effects
Accogli *et al*. (2019)*	–	15	No	Not explicitly stated	VH	not specified	Gradual improvement	NR
Bey *et al*. (2020)	Case 1 Case 2 Case 3 Case 4	13 14 12 Unclear	Yes Yes Yes No	No Yes Unclear Yes	AH, VH AH, VH AH, VH, paranoia no details provided	not specified aripiprazole (NR)^d^, olanzapine (NR)^d^, possibly others not specified risperidone (NR) clozapine (NR)^e^, others not specified	Poor Poor Temporary improvement Poor Improvement	NR NR NR NR NR
Boley *et al*. (2024)**	–	Unclear	Yes	Yes	no details provided	aripiprazole (NR), risperidone (NR), ziprasidone (NR), asenapine (NR), clozapine (NR), haloperidol (NR), quetiapine (1200 mg)^d^, olanzapine (2.5 mg)^d^	It is noted that various symptoms (including psychotic symptoms) returned when antipsychotics were stopped. Quetiapine and olanzapine in particular were noted to be partially effective.	‘minimal side effects’
Breckpot *et al*. (2016)	Patient 1 Patient 2	NR NR	Yes Yes	No No	‘unspecified nonorganic psychosis’ ’unspecified nonorganic psychosis’	NR NR	- -	- -
Denayer *et al*. (2012)	Patient 4	NR	Yes	Yes	no details provided	not specified	Poor	concern for NMS
De Rubeis *et al*. (2018)	S12	12–13	Yes	Yes	AH, VH	NR	–	–
Egger *et al*. (2016)***	Patient 2	∼ 22	No	Yes	unspecified hallucinations	haloperidol (NR) risperidone (NR)	NR NR	possible serotonin syndrome in combination with paroxetine
Fokstuen *et al*. (2016)	7	NR	No	No	no details provided	NR	–	–
Galosi *et al*. (2021)****	–	13	Yes	Yes	VH, ‘psychotic features’	none	–	–
Hu *et al*. (2020)	–	NR	NR	NR	AH, ‘bizarre delusion’	NR	–	–
Jungová *et al*. (2018)*****	–	23	Yes	Not explicitly stated, but patient diagnosed with ‘atypical bipolar disorder’	‘acute and transient psychotic disorder’, ‘atypical bipolar psychosis’, delusions	risperidone (NR) olanzapine (20 mg) quetiapine (NR) chlorprothixene (NR)^d^ haloperidol (NR)^d^	NR Good (before relapses) NR Good NR	EPS EPS, weight+ NR NR NR
Kankuri-Tammilehto *et al*. (2021)******	Sister Brother	36 15	No No	Yes Yes	no details provided no details provided	none risperidone (NR), haloperidol (NR)	- NR NR	- NR NR
Kohlenberg *et al*. (2020)	Case 10 Case 16 Case 19 Case 33 Case 36 Case 38	14? 13 Unclear 7 14 15	Yes Yes Yes Not explicitly stated Yes Yes	Yes Yes Yes Yes No Yes	paranoia paranoia no details provided ’bizarre behaviour with agitation, disorientation and insomnia’ paranoia ’talking to self’ (not explicitly characterised as being psychotic in nature, however)	NR NR NR NR NR NR	- - - - - -	- - - - - -
Li et al., (2016)	Case 2	NR	Yes	No	no details provided	not specified	Poor	NR
Messias *et al*. (2013)	–	32	Yes	Yes	No frank psychotic symptoms described, but her episode of confusion and bizarre behaviour was characterised as representing a psychotic episode and she carried diagnoses of ‘major depressive disorder with psychotic features’ and schizophrenia	quetiapine (600 mg)	Good	none reported
Rysstad *et al*. (2022)	–	‘late twenties’	Not explicitly stated	Yes	‘depressive delusions’ (belief that family members had died), hallucinations, disorganisation	risperidone (2 mg) aripiprazole (10 mg) olanzapine (15 mg)^d^	Not for psychosis Partial Used to treat mania	bradykinesia tremor none
Tabet *et al*. (2017)	11	NR	No	Yes	unspecified hallucinations	NR	–	–
Verhoeven *et al*. (2020)	Patient 1 Patient 2 Patient 16^a^	NR NR NR	Yes Yes Yes	Yes Yes Yes	‘atypical (cycloid) psychosis’ ‘manic psychosis’ ‘affective psychosis’	olanzapine (15 mg)^d^, others not specified none zuclopenthixol (NR), periciazine (NR), pimozide (NR), thioproperazine (NR), haloperidol (NR), thioridazine (NR), pipamperone (120 mg)	‘stabilization of mood and behaviour’ - NR NR NR NR NR NR NR	NR – NR NR NR NR NR NR NR
	Patient 18 Patient 20^b^ Patient 22^b^ Patient 23 Patient 24	NR NR NR NR NR	Yes No No Yes No	Yes Yes Yes Yes Yes	no details provided ‘manic psychosis’ no details provided previous diagnosis of ‘schizophrenia, disorganized subtype’ and diagnosed by the authors as having ‘schizoaffective disorder with catatonic features’ ‘recurrent psychoses with hypomanic features’	risperidone (NR) olanzapine (NR) quetiapine (600 mg)^d^ pipamperone (120 mg)^d^, others not specified quetiapine (1000 mg)^d^ olanzapine (2.5 mg), others not specified pipamperone (NR), sulpiride (NR), haloperidol (NR), risperidone (NR), levomepromazine (NR) olanzapine (10 mg)^d^	NR NR ‘stabilization of mood and behaviour’ ‘initially, relative amelioration of general functioning, later followed by slowly progressive deterioration’ ‘stabilization of mood and behaviour’ ‘spontaneous remission of catatonic features slowly progressive deterioration’ (with ECT?) NR NR NR NR NR ‘stable functioning’	NR NR NR NR NR NR NR NR NR NR NR NR

* this individual also had Turner syndrome.

** this individual also had a central nervous system BH_4_ deficiency.

*** this individual’s psychotic symptoms in retrospect were queried to have been related to an underlying delirium caused by a urinary tract infection.

**** this individual also harboured a *SYNJ1* variant classified as ‘likely pathogenic’ presumably accounting for her early-onset parkinsonism.

***** this individual’s psychiatric episodes were believed to be triggered by fever.

****** psychotic symptoms developed around the time that seizures recurred and both her seizures and psychosis resolved with benzodiazepine therapy.

a previously published in Verhoeven *et al*. (2013).

b previously published in Egger *et al*. (2016).

c does not include mood stabilizers or other treatments (e.g., forms of immunotherapy or electroconvulsive therapy)

d used in combination with mood stabilizer(s)

e this individual’s symptoms improved on clozapine but in combination with cyclophosphamide

AH = auditory hallucinations; VH = visual hallucinations; NR = not reported; ECT = electroconvulsive therapy; EPS = extrapyramidal symptoms

### Age of psychosis onset

The age of psychosis onset was only reported for 16 of the individuals in Table 1 (not including the patient described by Rysstad *et al*. (2022), whose age of onset was ambiguously described as being in his ‘late twenties’) in addition to four individuals described in a study by Gauthier *et al*. (2010) (however, this study was excluded from all additional analyses and from Table 1, given the limited clinical information otherwise provided). The average age of onset among these 20 individuals was ∼ 17 years, the range was 7-36 years, and 65% (13/20) developed psychotic symptoms at 15 years of age or earlier.

### Comorbid psychiatric features and phenomenology of psychosis

In cases where there was enough phenotypic information provided to surmise if comorbid catatonic and/or mood symptoms had occurred (i.e., either direct mention of such symptoms or inclusion of a suggestive diagnosis—e.g., a bipolar disorder diagnosis was sufficient to indicate the occurrence of comorbid mood symptoms for the purposes of this review), ∼69% (22/32) of individuals had also experienced catatonia at some point, ∼81% (26/32) had experienced mood symptoms, and 50% (16/32) had experienced both.

The type of psychotic symptom(s) that occurred was specified in only ∼46% (16/35) of patients, and few details were provided in most cases. The 16 individuals in question include those described by Accogli *et al*. (2019); De Rubeis *et al*. (2018); Egger *et al*. (2016); Galosi *et al*. (2021); Hu *et al*. (2020), Jungová *et al*. (2018), Messias *et al*. (2013), Rysstad *et al.* (2022), Tabet *et al*. (2017), and Patient 23 in the study by Verhoeven *et al*. (2020), as well as Cases 10, 16, and 36 described by Kohlenberg *et al*. (2020) and Cases 1, 2, and 3 described by Bey *et al*. (2020).

Of these individuals, ∼38% (6/16) experienced visual hallucinations (De Rubeis *et al*., 2018; Accogli *et al*., 2019; Bey *et al*., 2020; Galosi *et al*., 2021), ∼31% (5/16) experienced auditory hallucinations (De Rubeis *et al*., 2018; Bey *et al*., 2020; Hu *et al*., 2020), three additional individuals experienced unspecified hallucinations (Egger *et al*., 2016; Tabet *et al*., 2017; Rysstad *et al*., 2022), and ∼ 25% (4/16) experienced paranoia (Bey *et al*., 2020; Kohlenberg *et al*., 2020). Additional psychotic symptoms included bizarre (Hu *et al*., 2020), depressive (Rysstad *et al*., 2022), and unspecified (Jungova *et al*., 2018) delusions, as well as disorganisation in two individuals (Verhoeven *et al*., 2020; Rysstad *et al*., 2022).

Moreover, in addition to these 16 individuals, possible symptoms of psychosis were described for two other patients in the study by Kohlenberg *et al*. (2020) (Case 33 and 38). However, their symptoms (e.g., bizarre behaviour, disorientation, and self-talk) were not definitively frankly psychotic in nature (e.g., well characterised hallucinations or delusions). Nonetheless, these individuals were included in this review given that a brief psychotic episode was queried in the former’s case, while the latter individual was observed talking to herself in the context of psychiatrically decompensating (likely representing response to internal stimuli).

### Antipsychotic treatment response and tolerability

Information regarding antipsychotic treatment response was provided for only 17 individuals, and in many cases the corresponding descriptions were either vague and/or not specific to symptoms of psychosis. With these caveats in mind, ∼76% (13/17) of individuals at a minimum exhibited a partial and/or temporary response to at least one antipsychotic trialled (Messias *et al*., 2013; Jungova *et al*., 2018; Accogli *et al*., 2019; Bey *et al*., 2020; Verhoeven *et al*., 2020; Rysstad *et al*., 2022; Boley *et al*., 2024), whereas only four individuals were clearly reported to have had an exclusively poor response to antipsychotic therapy (Denayer *et al*., 2012, Li *et al*., 2016, Bey *et al*., 2020). A wide range of antipsychotics have been reported, with the two most common being olanzapine and risperidone, both having been used in eight cases. However, response to risperidone was described for only one individual whose symptoms were treatment refractory (Bey *et al*., 2020), whereas olanzapine was reported to be of at least some benefit in six cases (Jungova *et al*., 2018; Bey *et al*., 2020; Verhoeven *et al*., 2020; Boley *et al*., 2024). Specifically, as noted in Table 1, two individuals treated with olanzapine in the study by Verhoeven *et al*. (2020) were noted to have exhibited ‘stabilization of mood and behaviour’ and ‘stable functioning’, respectively. Boley *et al*. (2024) stated that regular olanzapine use ‘seemed to decrease the fluctuations’ of manic and psychotic symptoms ‘over time’, whereas Case 2 described by Bey *et al*. (2020) was reported to have gradually returned to ‘her neuropsychiatric baseline over 8 months’ in response to being switched from aripiprazole to olanzapine, in addition to being maintained on oral contraceptives and lamotrigine (after experiencing manic and psychotic symptoms, followed by depression, catatonia, and regression). Lastly, Jungová *et al*. (2018) reported that their patient was ‘cured by olanzapine, benzodiazepine, and maprotiline’. They also later noted that ‘therapy by olanzapine was continued with good antipsychotic effect’, before eventually being tapered due to side effects. Importantly however, response to olanzapine in the sixth case was specifically described in relation to catatonia, and possibly with the concurrent use of ECT (Verhoeven *et al*., 2020). The dose range of olanzapine across studies was 2.5 mg-20 mg, with both mean and median doses of 10 mg.

Quetiapine was reported to be of benefit in four cases (Messias *et al*., 2013; Verhoeven *et al*., 2020; Boley *et al*., 2024), with a dose range of 600–1200 mg, and a mean dose of 850 mg. The only other antipsychotics reported to have had some therapeutic benefit (in one case each) were clozapine (Bey *et al*., 2020), pipamperone (Verhoeven *et al*., 2020), chlorprothixene (Jungova *et al*., 2018), and aripiprazole (Rysstad *et al*., 2022).

Of the individuals who responded to one of these six antipsychotics, age at the time of treatment ranged from 14 to 52 years with a mean age of 33.9 years, excluding two individuals whose specific ages were not provided; however, both of whom were reported to be in their late 20s (Rysstad *et al*., 2022; Boley *et al*., 2024). Clinical details relevant to prescribing antipsychotic medications in the real world, such as weight/body mass index and vital sign information, were not provided in any of these articles.

The only antipsychotics specifically reported to have been ineffective (in one case each) were aripiprazole (Bey *et al*., 2020) and risperidone (Bey *et al*., 2020) (as mentioned); however, the doses used in these cases were not provided.

Side effects were reported in only four cases (Denayer *et al*., 2012; Egger *et al*., 2016; Jungova *et al*., 2018; Rysstad *et al*., 2022), and most notably involved concern for neuroleptic malignant syndrome in one individual (Denayer *et al*., 2012).

### Additional articles of relevance

Three additional articles of relevance were identified (Gauthier *et al*., 2010; Shaw *et al*., 2011; de Sena Cortabitarte *et al*., 2017); however, they were not included in Table 1 or in the main analyses, as they either provided very few phenotypic details or did not provide information in a patient-specific manner. The only exception was the inclusion of the study by Gauthier *et al*. (2010) in the age of onset calculations. Two of these studies sequenced schizophrenia spectrum disorder cohorts and identified a small number of individuals with *SHANK3* variants, but provided minimal clinical information (Gauthier *et al*., 2010; de Sena Cortabitarte *et al*., 2017). The other study, relying on parental reports, found high psychosis scores in children with Phelan-McDermid syndrome; however, no patient-specific data were provided (Shaw *et al*., 2011).

## Discussion

This review identified 18 articles that provided phenotypic information with respect to symptoms of psychosis in a patient-specific manner for 35 individuals who harboured genetic variants associated with Phelan-McDermid syndrome. A number of findings in particular deserve further discussion.

First, it is notable that so few published reports exist given that psychosis is a commonly cited feature of Phelan-McDermid syndrome (Srivastava *et al*., 2023). Moreover, not only is the absolute number of published reports low, but many of which provide very limited clinical information (at least with respect to symptoms of psychosis). For example, information regarding the type of psychotic symptoms that occurred, as well as details pertaining to antipsychotic treatment response, were available for less than half of all cases, with often vague and minimally detailed descriptions provided. Nonetheless, ∼76% of individuals whose response to treatment was described exhibited at least partial and/or temporary improvement in relation to a variety of antipsychotic medications, suggesting that antipsychotic therapy may be reasonably efficacious for symptoms of psychosis in Phelan-McDermid syndrome. In particular, olanzapine was found to be of benefit for six individuals at typical (or even low) doses (Jungova *et al*., 2018; Bey *et al*., 2020; Verhoeven *et al*., 2020; Boley *et al*., 2024) and there were no reports of olanzapine non-response. Similarly, quetiapine was reported to be of benefit in four cases (Messias *et al*., 2013; Verhoeven *et al*., 2020; Boley *et al*., 2024) and there were no reports of quetiapine non-response; however, supratherapeutic doses were used for two of the four individuals (1000 mg and 1200 mg) (Verhoeven *et al*., 2020; Boley *et al*., 2024), potentially indicating a suboptimal response to lower doses. Clozapine was used in two patients (Bey *et al*., 2020; Boley *et al*., 2024) but treatment response was described for only one, and although this individual’s symptoms improved, they were also undergoing concurrent cyclophosphamide therapy, confounding the interpretation of matters (Bey *et al*., 2020).

Importantly, despite these arguably encouraging findings, the possible benefits of antipsychotic therapy must be weighed against the potential for inducing catatonia in Phelan-McDermid syndrome, such that current guidelines recommend that doses be kept low (Srivastava *et al*., 2023) and that the use of antipsychotic monotherapy be approached with caution (van Balkom *et al*., 2023). Perhaps surprisingly, only one article included in this review explicitly commented on a possible relationship between the use of antipsychotics and the development of catatonia in their respective patients (Kohlenberg *et al*., 2020). Moreover, the authors of this study did not provide patient-specific information in this respect but noted that seven of the 20 individuals in their cohort who experienced catatonia were on neuroleptics at the time of catatonic symptom onset (however, it is not clear how many of these individuals experienced psychosis). For only two other individuals included in this review is it made clear that antipsychotic treatment preceded the development of catatonic symptoms, such that the use of antipsychotic medication could have conceivably contributed (Case 2 in the study by Bey *et al*. (2020) and Patient 4 in the study by Denayer *et al*. (2012)); however, specific timeline details strongly supporting this possibility in these cases are lacking. Otherwise, numerous of the individuals described by Verhoeven *et al*. (2020) included in this review had a lifetime history of both catatonia and exposure to antipsychotics, making a relationship between the two possible, but entirely speculative. Although not specific to Phelan-McDermid syndrome, it is worth noting that higher potency antipsychotics are thought to carry a greater risk of inducing catatonia, and as such the use of atypical antipsychotics with a lower affinity for D2 receptors, including olanzapine and quetiapine, may be preferable in individuals with, or at risk for catatonia, when antipsychotic therapy is indicated (Smith and Holmes, 2023).

Another interesting finding is how infrequently side effects were noted to have occurred in general, particularly given that intellectual disability populations are known to be sensitive to certain side effects of antipsychotic medications (Sheehan *et al*., 2017). However, in the vast majority of cases there was simply no mention of side effects, rather than an explicit statement that no side effects occurred or that antipsychotic therapy was well tolerated. As such, in some instances the occurrence of side effects may have conceivably been omitted from the reports.

Phenomenologically, more individuals experienced visual hallucinations than any other particular psychotic symptom, including auditory hallucinations or paranoia. Moreover, visual hallucinations were the only psychotic symptom reported in two cases (Accogli *et al*., 2019; Galosi *et al*., 2021), suggesting that the nature of psychotic presentations in Phelan-McDermid syndrome (at least in a subset of patients) may be qualitatively different than in general schizophrenia populations, given that the occurrence of visual hallucinations in isolation is unusual for schizophrenia. However, it is important to note that one of these individuals also had an early-onset form of Parkinson’s disease, which may have accounted for the development of visual hallucinations (Galosi *et al*., 2021).

The age of onset was also much younger in many cases than is typical of schizophrenia or any other primary psychotic disorder, arguably further distinguishing Phelan-McDermid syndrome-associated psychosis from ‘idiopathic’ schizophrenia.

Another notable but perhaps unsurprising finding is that the majority of individuals also experienced either catatonic or mood symptoms (often bipolar disorder-like presentations), and half experienced both. This further distinguishes Phelan-McDermid syndrome-associated psychosis from the typical phenotype of schizophrenia, as although estimates vary, far less than half of individuals with ‘idiopathic’ schizophrenia experience catatonia (Walther and Strik, 2016), and schizophrenia by definition is not associated with a significant burden of mood symptoms. As such, bipolar 1 or 2 disorder with psychotic features or schizoaffective disorder are presumably more applicable diagnoses for the majority of patients with Phelan-McDermid syndrome when psychotic symptoms are present. Nonetheless, given that no mood symptoms were explicitly reported in ∼19% of cases, typical schizophrenia-like presentations may still be possible (the presence of any comorbid neurodevelopmental abnormalities notwithstanding). Arguing against this, however, is that age of onset was reported for only two individuals without a history mood symptoms (Bey *et al*., 2020; Kohlenberg *et al*., 2020), and in both cases it was atypically early for schizophrenia (13 and 14 years of age) (Lewine and Hart, 2020).

This review had a number of limitations that seriously confound interpretation of the results, not the least of which being how few reports have been published. Moreover, the available information is largely anecdotal, having come from case reports or relatively small case series. Additionally, many of the reports provided very limited and often vague clinical information with respect to symptoms of psychosis. For example, regarding treatment response, although general statements were sometimes made to describe improvements in behaviour, mood, and/or functioning, it was often difficult to delineate the therapeutic effects of antipsychotic therapy on the individuals’ symptoms of psychosis, specifically. Further confounding the assessment thereof, in eight cases antipsychotic therapy was used in combination with a mood stabilizer (Jungova *et al*., 2018; Bey *et al*., 2020; Verhoeven *et al*., 2020; Boley *et al*., 2024), making it impossible to disentangle the individual therapeutic effects of each. This is particularly problematic given that all eight of these individuals were considered treatment responders, meaning that ∼62% (8/13) of those who benefitted from antipsychotic therapy were also taking a mood stabilizer; however, in one of these cases olanzapine was initially reported to have had a ‘good antipsychotic effect’ in the absence of a mood stabilizer (before this individual later relapsed, prompting the addition of valproic acid among other medications) (Jungova *et al*., 2018). Similarly, one of the other individuals who responded to antipsychotic therapy was concurrently treated with cyclophosphamide (Bey *et al*., 2020) and another may have received ECT (Verhoeven *et al*., 2020). This caveat particularly calls into question the efficacy of both olanzapine and quetiapine in in this context, given that of the six individuals who responded to olanzapine, four were also taking a mood stabilizer (Bey *et al*., 2020; Verhoeven *et al*., 2020; Boley *et al*., 2024) and one may have received ECT (Verhoeven *et al*., 2020), and of the four individuals who responded to quetiapine, three were taking a mood stabilizer (Verhoeven *et al*., 2020; Boley *et al*., 2024). As such, the degree to which antipsychotic therapy, specifically, contributed to symptom improvement in these cases remains unclear.

The results with respect to treatment response in particular may also have been influenced by a positive publication bias. Similarly, it is possible that treatment response was poorer among the individuals described in the reports that mentioned the use of antipsychotics but that did not provide any information pertaining to their effectiveness.

Lastly, as noted by Shaw *et al*. (2011), correctly diagnosing psychotic symptoms in Phelan-McDermid syndrome may be difficult given the broader developmental phenotype of this population, and similar concerns have been raised with respect to other genetic neurodevelopmental disorders (Colijn and Stowe, 2024). In particular, the common occurrence of intellectual disability in Phelan-McDermid syndrome may impact an individual’s capacity to reliably describe their inner experiences, especially considering the high prevalence of significant speech/language impairment, including a total absence of speech in some cases (Burdeus-Olavarrieta *et al*., 2023). Moreover, numerous autistic behaviours can superficially resemble response to internal stimuli and other psychosis-mediated clinical observations. In my own experience as a clinician, differentiating between child-like retreat into fantasy and frank psychosis can be a difficult task in Phelan-McDermid syndrome, particularly given the numerous aforementioned confounding variables potentially at play. Perhaps the most helpful distinguishing feature to consider is the evolution (or lack thereof) of such symptoms longitudinally, relative to an individual’s psychiatric baseline. With this in mind, at a minimum treatment should be considered when query psychotic symptoms, as a result of having qualitatively changed over time, become more prominent, distressing, and/or functionally impairing. However, the choice of treatment may in part depend on the presence or absence of other neuropsychiatric and medical issues, and should conform, where possible, to recommendations from existing guidelines (Srivastava *et al*., 2023; van Balkom *et al*., 2023). More broadly speaking, the results of this review in general should be interpreted in the wider context of these papers’ recommendations, rather than viewed as a replacement for them.

Given the relatively few reports of psychosis occurring in Phelan-McDermid syndrome, it is recommended that clinicians who have assessed and managed symptoms of psychosis in this population consider publishing their clinical experiences in this respect (at a minimum individual case reports, but ideally larger case series or cohort data). Information that will bolster the usefulness of future reports in this context include psychosis age of onset, the cooccurrence and temporal association between psychotic symptoms and other clinical features (in particular, catatonic and mood symptoms), the temporal association between the development of catatonia and the initiation of antipsychotic therapy, relevant laboratory/imaging/electrophysiology results to rule out contributory medical issues, and of course a robust description of the psychotic symptoms, as well as the authors’ rationale for excluding non-psychotic explanations for such symptoms. With respect to treatment, it is imperative that the doses employed (and resultant blood levels where possible), therapeutic response, the occurrence of side effects, and the use of concurrent psychotropic medications, be systematically described.

## Conclusion

This systematic review perhaps most importantly highlights the paucity of high-quality clinical data pertaining to the occurrence of psychotic symptoms in Phelan-McDermid syndrome. The findings nonetheless suggest that although psychotic features occur in a subset of affected individuals, the nature of such presentations may qualitatively differ from that observed in general schizophrenia populations. In particular, psychotic symptoms may have an earlier age of onset in Phelan-McDermid syndrome and may be more likely to occur in combination with catatonic and mood symptoms. Visual hallucinations may also be more common (in general and possibly specifically in the absence of auditory hallucinations).

Additionally, although antipsychotic medications, in particular olanzapine and quetiapine, may be effective in treating psychotic symptoms in this population, the corresponding evidence is anecdotal, based on a very small number of reports, and confounded by the frequent concurrent use of various mood stabilizers. As such, this review highlights the need for more published reports with detailed phenotypic descriptions, including with respect to antipsychotic treatment response, before any firm conclusions can be drawn. With this in mind, clinicians should continue to make treatment decisions in this context in accordance with published guidelines that address the management of other common neuropsychiatric symptoms in Phelan-McDermid syndrome.

## Data Availability

NA

## References

[ref1] Accogli A , Yang R , Blain-Juste ME , Braverman N , Shah J and Trakadis Y (2019) SHANK3 mutation and mosaic turner syndrome in a female patient with intellectual disability and psychiatric features. The Journal of Neuropsychiatry and Clinical Neurosciences 31, 272–275. 30888922 10.1176/appi.neuropsych.18100228

[ref2] Bey AL , Gorman MP , Gallentine W , Kohlenberg TM , Frankovich J , Jiang YH and Van Haren K (2020) Neuropsychiatric Syndrome in Girls With SHANK3 Mutations Responds to Immunomodulation. Pediatrics 145 10.1542/peds.2019-1490 PMC7802010 32015180

[ref3] Boley G , Pierri J , Finegold D and Pan L (2024) Evaluation of catatonia in autism and severe depression revealing phelan-mcDermid syndrome and tetrahydrobiopterin deficiency. British medical journal Case Reports 17, e256155. 10.1136/bcr-2023-256155 PMC10773351 38176751

[ref4] Breckpot J , Vercruyssen M , Weyts E , Vandevoort S , D’Haenens G , Van Buggenhout G , Leempoels L , Brischoux-Boucher E , Van Maldergem L , Renieri A , Mencarelli MA , D’Angelo C , Mericq V , Hoffer MJ , Tauber Mé , Molinas C , Castiglioni C , Brison N , Vermeesch JR , Danckaerts M , Sienaert P , Devriendt K , Vogels A (2016) Copy number variation analysis in adults with catatonia confirms haploinsufficiency of SHANK3 as a predisposing factor. European Journal of Medical Genetics 59, 436–443. 27519580 10.1016/j.ejmg.2016.08.003

[ref5] Burdeus-Olavarrieta M , Nevado J , Van Weering-Scholten S , Parker S , European Phelan-Mcdermid Syndrome C and Swillen A (2023) Consensus recommendations on communication, language and speech in phelan-mcDermid syndrome. European Journal of Medical Genetics 66, 104745. 36871884 10.1016/j.ejmg.2023.104745

[ref6] Colijn MA and Stowe RM (2024) The curious absence of psychosis in GRIN1-related neurodevelopmental disorder. European archives of psychiatry and clinical neuroscience (Online ahead of print). 10.1007/s00406-024-01796-x 38483682

[ref7] De Rubeis S , Siper PM , Durkin A , Weissman J , Muratet F , Halpern D , Trelles MDP , Frank Y , Lozano R , Wang AT , Holder JL, Jr., Betancur C , Buxbaum JD , Kolevzon A (2018) Delineation of the genetic and clinical spectrum of phelan-mcDermid syndrome caused by SHANK3 point mutations. Molecular Autism 9, 31. 29719671 10.1186/s13229-018-0205-9 PMC5921983

[ref8] De Sena Cortabitarte A , Degenhardt F , Strohmaier J , Lang M , Weiss B , Roeth R , Giegling I , Heilmann-Heimbach S , Hofmann A , Rujescu D , Fischer C , Rietschel M , Nothen MM , Rappold GA and Berkel S (2017) Investigation of SHANK3 in schizophrenia. American Journal of Medical Genetics Part B: Neuropsychiatric Genetics 174, 390–398. 10.1002/ajmg.b.32528 28371232

[ref9] Denayer A , Van Esch H , De Ravel T , Frijns JP , Van Buggenhout G , Vogels A , Devriendt K , Geutjens J , Thiry P and Swillen A (2012) Neuropsychopathology in 7 Patients with the 22q13 deletion syndrome: presence of bipolar disorder and progressive loss of skills. Molecular Syndromology 3, 14–20. 22855650 10.1159/000339119 PMC3398818

[ref10] Dille Y , Lagae L , Swillen A and Buggenhout GV (2023) Neurodevelopmental profile and stages of regression in phelan-mcDermid syndrome. Developmental Medicine and Child Neurology 65, 917–925. 36477723 10.1111/dmcn.15482

[ref11] Egger JI , Zwanenburg RJ , Van Ravenswaaij-Arts CM , Kleefstra T and Verhoeven WM (2016) Neuropsychological phenotype and psychopathology in seven adult patients with phelan-mcDermid syndrome: implications for treatment strategy. Genes, Brain and Behavior 15, 395–404. 26824576 10.1111/gbb.12285

[ref12] Fokstuen S , Makrythanasis P , Hammar E , Guipponi M , Ranza E , Varvagiannis K , Santoni FA , Albarca-Aguilera M , Poleggi ME , Couchepin F , Brockmann C , Mauron A , Hurst SA , Moret C , Gehrig C , Vannier A , Bevillard J , Araud T , Gimelli S , Stathaki E , Paoloni-Giacobino A , Bottani A , Sloan-Bena F , Sizonenko LD , Mostafavi M , Hamamy H , Nouspikel T , Blouin JL and Antonarakis SE (2016) Experience of a multidisciplinary task force with exome sequencing for mendelian disorders. Human Genomics 10, 24. 27353043 10.1186/s40246-016-0080-4 PMC4924303

[ref13] Galosi S , Martinelli S , Pannone L , Terrinoni A , Venditti M , Pizzi S , Ciolfi A , Chillemi G , Gigliotti F , Cesario S , Tartaglia M and Leuzzi V (2021) Co-occurring SYNJ1 and SHANK3 variants in a girl with intellectual disability, early-onset parkinsonism and catatonic episodes. Parkinsonism & Related Disorders 84, 5–7. 33515856 10.1016/j.parkreldis.2020.12.022

[ref14] Gauthier J , Champagne N , Lafrenière RG , Xiong L , Spiegelman D , Brustein E , Lapointe M , Peng H , Côté Mélanie , Noreau A , Hamdan FF , Addington Aé M , Rapoport JL , DeLisi LE , Krebs M-O , Joober R , Fathalli F , Mouaffak Fçal , Haghighi AP , Néri C , Dubé M-P , Samuels ME , Marineau C , Stone EA , Awadalla P , Barker PA , Carbonetto S , Drapeau P , Rouleau GA , Diallo O , Duguay J , Drits M , Henrion E , Jolivet P , Kuku Fédéric , Lachapelle K , Laliberté G , Laurent S , Liao M , Marino C , Piton Aélie , Raymond A , Reynolds A , Rochefort D , St-Onge J , Thibodeau P , Tsurudome K , Yang Y , Leroy S , Ossian K , Chayet Mélanie , Gourion D (2010) De novo mutations in the gene encoding the synaptic scaffolding protein SHANK3 in patients ascertained for schizophrenia. Proceedings of the National Academy of Sciences 107, 7863–7868. 10.1073/pnas.0906232107 PMC2867875 20385823

[ref15] Hu TM , Wang YC , Wu CL , Hsu SH , Tsai HY and Cheng MC (2020) Multiple rare risk coding variants in postsynaptic density-related genes associated with schizophrenia susceptibility. Frontiers in Genetics 11, 524258. 33343614 10.3389/fgene.2020.524258 PMC7746813

[ref16] Huang YS , Fang TH , Kung B and Chen CH (2022) Two genetic mechanisms in two siblings with intellectual disability, autism spectrum disorder, and psychosis. Journal of Personalized Medicine 12, 1013. 35743796 10.3390/jpm12061013 PMC9224546

[ref17] Jungova P , Cumova A , Kramarova V , Lisyova J , Durina P , Chandoga J and Bӧhmer D (2018) Phelan-mcDermid syndrome in adult patient with atypical bipolar psychosis repeatedly triggered by febrility. Neurocase 24, 227–230. 30376408 10.1080/13554794.2018.1542007

[ref18] Kankuri-Tammilehto M , Sauna-Aho O and Arvio M (2021) Neurocognitive follow-up in adult siblings with phelan-mcDermid syndrome due to a novel SHANK3 splicing site mutation. Molecular Genetics & Genomic Medicine 9, e1780. 34369668 10.1002/mgg3.1780 PMC8683620

[ref19] Kohlenberg TM , Trelles MP , Mclarney B , Betancur C , Thurm A and Kolevzon A (2020) Psychiatric illness and regression in individuals with phelan-mcDermid syndrome. Journal of Neurodevelopmental Disorders 12, 7. 32050889 10.1186/s11689-020-9309-6 PMC7014655

[ref20] Kolevzon A , Delaby E , Berry-Kravis E , Buxbaum JD and Betancur C (2019) Neuropsychiatric decompensation in adolescents and adults with phelan-mcDermid syndrome: a systematic review of the literature. Molecular Autism 10, 50. 31879555 10.1186/s13229-019-0291-3 PMC6930682

[ref21] Levy T , Foss-Feig JH , Betancur C , Siper PM , Trelles-Thorne MDP , Halpern D , Frank Y , Lozano R , Layton C , Britvan B , Bernstein JA , Buxbaum JD , Berry-Kravis E , Powell CM , Srivastava S , Sahin M , Soorya L , Thurm A , Kolevzon A and Developmental Synaptopathies C (2022) Strong evidence for genotype-phenotype correlations in phelan-mcDermid syndrome: results from the developmental synaptopathies consortium. Human Molecular Genetics 31, 625–637. 34559195 10.1093/hmg/ddab280 PMC8863417

[ref22] Lewine R and Hart M (2020) Schizophrenia spectrum and other psychotic disorders. Handbook of clinical neurology 175, 315–333. 33008535 10.1016/B978-0-444-64123-6.00022-9

[ref23] Li EH , Stork CE , Jim On SC , Bryson EO , Aloysi AS and Kellner CH (2016) Additional procedures performed during electroconvulsive therapy anesthesia. The Journal of ECT 32, e7–8. 26669745 10.1097/YCT.0000000000000288

[ref24] Messias E , Kaley SN and Mckelvey KD (2013) Adult-onset psychosis and clinical genetics: a case of phelan-mcDermid syndrome. The Journal of Neuropsychiatry and Clinical Neurosciences 25, E27. 10.1176/appi.neuropsych.12100241 PMC4096959 24247879

[ref25] Rysstad AL , Kildahl AN , Skavhaug JO , Donnum MS and Helverschou SB (2022) Case study: organizing outpatient pharmacological treatment of bipolar disorder in autism, intellectual disability and phelan-mcDermid syndrome (22q13.3 deletion syndrome). International Journal of Developmental Disabilities 68, 378–387. 35603006 10.1080/20473869.2020.1756113 PMC9122368

[ref26] Shaw SR , Rahman A and Sharma A (2011) Behavioral profiles in phelan-mcDermid syndrome: focus on mental health. Journal of Mental Health Research in Intellectual Disabilities 4, 1–18.

[ref27] Sheehan R , Horsfall L , Strydom Aé , Osborn D , Walters K and Hassiotis A (2017) Movement side effects of antipsychotic drugs in adults with and without intellectual disability: UK population-based cohort study. British medical journal open 7, e017406. 10.1136/bmjopen-2017-017406 PMC5724123 28775195

[ref28] Smith AC and Holmes EG (2023) Catatonia: a narrative review for hospitalists. American Journal of Medicine Open 10, 100059. 39035239 10.1016/j.ajmo.2023.100059 PMC11256243

[ref29] Srivastava S , Sahin M , Buxbaum JD , Berry-Kravis E , Soorya LV , Thurm A , Bernstein JA , Asante-Otoo A , Bennett WE Jr. , Betancur C , Brickhouse TH , Passos Bueno MR , Chopra M , Christensen CK , Cully JL , Dies K , Friedman K , Gummere B , Holder JL Jr. , Jimenez-Gomez A , Kerins CA , Khan O , Kohlenberg T , Lacro RV , Levi LA , Levy T , Linnehan D , Eva L , Moshiree B , Neumeyer A , Paul SM , Phelan K , Persico A , Rapaport R , Rogers C , Saland J , Sethuram S , Shapiro J , Tarr PI , White KM , Wickstrom J , Williams KM , Winrow D , Wishart B and Kolevzon A (2023) Updated consensus guidelines on the management of phelan-mcDermid syndrome. American Journal of Medical Genetics Part A 191, 2015–2044. 37392087 10.1002/ajmg.a.63312 PMC10524678

[ref30] Tabet AC , Rolland T , Ducloy M , Levy J , Buratti J , Mathieu A , Haye D , Perrin L , Dupont C , Passemard S , Capri Y , Verloes A , Drunat S , Keren B , Mignot C , Marey I , Jacquette A , Whalen S , Pipiras E , Benzacken B , Chantot-Bastaraud S , Afenjar A , Heron D , Le Caignec C , Beneteau C , Pichon O , Isidor B , David A , El Khattabi L , Kemeny S , Gouas L , Vago P , Mosca-Boidron AL , Faivre L , Missirian C , Philip N , Sanlaville D , Edery P , Satre V , Coutton C , Devillard F , Dieterich K , Vuillaume ML , Rooryck C , Lacombe D , Pinson L , Gatinois V , Puechberty J , Chiesa J , Lespinasse J , Dubourg C , Quelin C , Fradin M , Journel H , Toutain A , Martin D , Benmansour A , Leblond CS , Toro R , Amsellem F , Delorme R and Bourgeron T (2017) A framework to identify contributing genes in patients with phelan-mcDermid syndrome. npj Genomic Medicine 2, 32. 29263841 10.1038/s41525-017-0035-2 PMC5677962

[ref31] Van Balkom IDC , Burdeus-Olavarrieta M , Cooke J , De Cuba AG , Turner A , European Phelan-Mcdermid Syndrome C , Vogels A and Maruani A (2023) Consensus recommendations on mental health issues in phelan-mcDermid syndrome. European Journal of Medical Genetics 66, 104770. 37085014 10.1016/j.ejmg.2023.104770

[ref32] Verhoeven WM , Egger JI , Cohen-Snuijf R , Kant SG and De Leeuw N (2013) Phelan-mcDermid syndrome: clinical report of a 70-year-old woman. American Journal of Medical Genetics Part A 161A, 158–161. 23166010 10.1002/ajmg.a.35597

[ref33] Verhoeven WMA , Egger JIM and De Leeuw N (2020) A longitudinal perspective on the pharmacotherapy of 24 adult patients with phelan mcDermid syndrome. European Journal of Medical Genetics 63, 103751. 31465867 10.1016/j.ejmg.2019.103751

[ref34] Walther S and Strik W (2016) Catatonia. CNS spectrums 21, 341–348. 27255726 10.1017/S1092852916000274

